# CD64 Expression on Neutrophils (nCD64) as a Biomarker in Adult Patients With Sepsis: A Cross-Sectional Study

**DOI:** 10.7759/cureus.71912

**Published:** 2024-10-20

**Authors:** Rahul Kanungo, Surekha B Hippargi

**Affiliations:** 1 Pathology, Shri B.M. Patil Medical College, Hospital and Research Centre, Bijapur Lingayat District Educational Association (BLDE) (Deemed to be University), Vijayapura, IND

**Keywords:** cd64, c-reactive protein, flow cytometry, neutrophil, organ failure from sepsis, positive blood culture, quick sequential organ failure assessment (qsofa), sepsis, severe sepsis, total leukocyte count

## Abstract

Introduction

Neutrophils that are at rest exhibit very low expression of CD64, the high-affinity immunoglobulin fragment crystallizable γ receptor I, which is also found in monocytes. Neutrophils that have been exposed to endotoxins or are infected express more CD64.

Objectives

The aim of this study was to assess the CD64 biomarker expression on neutrophils using the flow cytometry method and its comparison with total leukocyte count, C-reactive protein (CRP) level, and blood culture sensitivity test in the diagnosis of sepsis in adults.

Materials and methods

Using the quick Sequential Organ Failure Assessment (qSOFA) scoring criteria, 94 blood samples from individuals with clinical indications of sepsis were included in this investigation. Samples were collected in K2 EDTA (ethylenediaminetetraacetic acid) vacutainers and analyzed for neutrophil CD64 (nCD64) levels using BD FACSLyric^TM^ flow cytometer (BD, Franklin Lakes, NJ) within 24 hours of collection. Total leukocyte count, CRP levels, and blood culture sensitivity tests were also assessed simultaneously.

Results

Majority of the patients, i.e., 29 (30.9%), belonged to the age group of 19 to 35 years, with a female preponderance. The mean total leukocyte count was 19.49 ± 8.12 x 10^3^/µL, mean CRP level was 81.19 ± 56.33 mg/dL, and the mean nCD64 expression - median fluorescence intensity was 197.26 ± 79.56. A positive blood culture was found in 59 (62.8%) cases. Neutrophils showed bright expression of nCD64 in 39 (41.5%) patients, dim expression in 35 (37.2%) patients, and moderate expression in 20 (21.3%) patients in the present study. The correlation between nCD64 expression with CRP, total leukocyte count, and blood culture sensitivity test was statistically significant (p=0.001). nCD64 expression showed a sensitivity of 93.2% and specificity of 91.4% for diagnosing sepsis, with an area under the curve (AUC) of 0.973. This proves nCD64 to be a highly effective biomarker compared to conventional methods.

Conclusion

nCD64 has a higher sensitivity and specificity as compared to total leukocyte count, CRP levels, and blood culture sensitivity test in the diagnosis of sepsis. It is an effective and rapid test that can enhance sepsis diagnostic accuracy, when combined with other biomarkers. Multicentric research needs to be conducted to validate these findings.

## Introduction

Bacterial infection is one of the most common clinical diseases [1]. Since 1928, antibiotic therapy has been regarded as the most effective treatment for infections. Bacterial culture sensitivity tests take a long time, which very often results in delay or wrong diagnosis [2].

Microbiological culture remains the gold standard for diagnosing sepsis, but it is time-consuming and has a risk of false negatives [3,4]. Several factors, such as the initiation of empirical antibiotic therapy before sample collection, can influence the results [5]. These challenges have led to the exploration of biomarkers for the early detection of sepsis. Recently, soluble biomarkers in serum or plasma have gained attention for their potential in improving infection diagnostics. Tumor necrosis factor, interleukins, procalcitonin (PCT), and neutrophil CD64 (nCD64) expressions are among the biomarkers being extensively studied [6]. nCD64, in particular, shows promise as a diagnostic parameter for systemic acute inflammatory responses or sepsis [7,8].

In current practice, identifying bacterial infections often involves detecting CD64 expression on peripheral neutrophils using flow cytometry. CD64, a high-affinity immunoglobulin fragment crystallizable γ receptor I (FcγRI) of human immunoglobulin G (IgG), is naturally expressed on macrophages, monocytes, and eosinophils [9]. In healthy individuals, neutrophils exhibit low levels of CD64. However, in those with bacterial infections, CD64 expression on neutrophils can increase significantly (over 10-fold) within hours, facilitating the distinction between resting and activated neutrophils [10]. Several studies have demonstrated that assessing nCD64 expression provides increased infection sensitivity and specificity. The nCD64 median fluorescence intensity (MFI) notably rises in bacterial infections, including sepsis, systemic infection, bronchitis, and bacterial peritonitis [11,12].

Neutrophils have surface receptors that recognize bacterial antigens, leading to their activation and subsequent phagocytosis. This process is facilitated by IgG receptors on neutrophils [13]. Several studies have identified nCD64 expression as a promising laboratory indicator for detecting sepsis and infection [14,15]. As immature myeloid cells develop into segmented neutrophils, their high expression of CD64 declines. CD64 expression on neutrophil surfaces is low in healthy persons, is stored in intracellular fluid, and is stimulated by both internal and external stimuli. Its expression in neutrophils can be induced by specific inflammatory cytokines and bacterial products in the cell wall [15].

Despite advances in sepsis diagnosis, traditional methods such as blood culture are time-consuming and often result in delayed treatment. This study aims to investigate the efficacy of nCD64 as a rapid, reliable biomarker for early sepsis detection. The investigation was conducted using flow cytometry.

Objective of the study

This study aims to assess nCD64 as a biomarker for sepsis and compare its diagnostic accuracy with total leukocyte count (TLC), C-reactive protein (CRP) levels, and blood culture sensitivity in adult patients.

## Materials and methods

Study design

A cross-sectional study involving 94 adult patients was conducted over 18 months, from September 1, 2022, to February 29, 2024, at Shri B.M. Patil Medical College, Hospital and Research Centre, Bijapur Lingayat District Educational Association (BLDE) (Deemed to be University), Vijayapura, Karnataka, India, following approval from the Institutional Ethical Committee of BLDE (Deemed to be University), obtained on August 30, 2022, under approval letter number BLDE(DU)/IEC/677/2022-23.

Population

Patients were included based on clinical suspicion of sepsis and excluded if they had a history of hematological malignancy, diabetes mellitus, rheumatoid arthritis, or chronic liver disease.

Methods of data collection

The parameters analyzed in this study were nCD64, TLC, CRP, and blood culture sensitivity test. Blood samples were collected in ethylenediaminetetraacetic acid (EDTA) tubes to prevent clotting and ensure accurate measurement of CD64 expression. Analysis for TLC was conducted using Sysmex-XN-1000™ Hematology Analyzer (Sysmex Inc., Lincolnshire, IL), and nCD64 expression was assessed using the BD FACSLyric™ flow cytometer (BD, Franklin Lakes, NJ) within 24 hours of collection.

The samples of the same patients were then sent for CRP level and blood culture sensitivity tests. On admission, all patients clinically suspected of having sepsis were evaluated for nCD64 using flow cytometry, TLC, CRP, and blood culture sensitivity test, along with general physical examination and systemic examination.

Antibody titration and analysis procedure

Two milliliters (mL) of EDTA anti-coagulated freshly collected human peripheral blood was taken. The total staining volume and number of cells in the final multicolor experiment was determined. Here, we assumed around a million cells in 100µL + 50µL of antibody cocktail, i.e., total staining volume of 150µL.

Serial dilutions of the CD64 antibody were prepared to ensure accurate quantification of nCD64 expression through flow cytometry. This method allows for the differentiation between dim, moderate, and bright expression levels, which are critical for diagnosing sepsis. The recommended volume for the antibody serum was 20µL per test. Stain buffer was added to tubes, as indicated in Table 1 (50µL to each tube except tube 1), and then 40µL of the CD64 allophycocyanin antibody was added to tube 1 in the series and mixed well. Following this, 50µL aspiration from tube 1 was done, added to tube 2, and mixed, and then this procedure was repeated in subsequent tubes (Table 1). Tube 7 had no antibody (unstained).

**Table 1:** nCD64 antibody titration procedure. The recommended volume for the antibody serum was 20µL per test. Ab, antibody; NA, not applicable

	Stain buffer	Transfer from the previous tube	Temporary volume	Transfer to the next tube	Final Ab-mix volume	Ab (µL)
Tube 1	60µL	40µL	100µL	50µL	50µL	20.00
Tube 2	50µL	50µL	100µL	50µL	50µL	10.00
Tube 3	50µL	50µL	100µL	50µL	50µL	5.00
Tube 4	50µL	50µL	100µL	50µL	50µL	2.50
Tube 5	50µL	50µL	100µL	50µL	50µL	1.25
Tube 6	50µL	50µL	100µL	50µL	50µL	0.6250
Tube 7	50µL	0µL	50µL	NA	50µL	0.0000

Sample preparation was done using the stain-lyse-wash surface staining protocol. Only a single antibody was used in our study, i.e., CD64.

Gating of neutrophils and nCD64 assessment

The granulocytes, including neutrophils, were identified based on their higher forward scatter and side scatter properties compared to other leukocytes, and a gate was manually drawn around this population. The granulocytes were further examined by creating a new plot from the previously gated population. A final gate was drawn around the neutrophils based on the scatter properties. We performed data analysis and plotting signal/noise graph and measured the nCD64 MFI. MFI refers to the average brightness of the fluorescent signal emitted by stained neutrophils, which correlates with the expression level of CD64 on their surface.

Assessment of other variables

Total leukocyte count (TLC) for the same samples were measured and recorded using Sysmex XN-1000 six-part automated hematology analyzer. After assessing the TLC, we next evaluated CRP levels. Additional blood samples were collected in EDTA vacutainers and sodium polyanethol sulfonate vacutainers and sent for TLC, CRP, and automated blood culture analysis, and the corresponding results were recorded.

Statistical analysis

Data entered into Microsoft Excel sheet (Microsoft Corp., Redmond, WA) were analyzed using SPSS Version 20 (IBM Corp., Armonk, NY). nCD64 levels were compared to the culture-negative patients to determine the degree of expression. Pearson's correlation was employed to assess the relationship between nCD64 expression and other variables, with a significance level set at p<0.05. The receiver operator characteristics (ROC) curve was plotted, and nCD64 sensitivity and specificity were measured.

## Results

The minimum and maximum ages in our study were 19 and 87 years, respectively. The mean age was 47.82 ± 17.99 years. Of the total of 94 patients, 29 (30.9%) patients were in the age group of 19-35 years, 25 (25.5%) patients in the age group of 36-50 years, 25 (25.5%) patients in the age group of 51-65 years, and 17 (18.1%) patients in the age group of more than 65 (>65) years (Table 2).

**Table 2:** Patient distribution on the basis of age groups.

Age groups	Frequency (%)
19 to 35 years	29 (30.9)
36 to 50 years	24 (25.5)
51 to 65 years	24 (25.5)
>65 years	17 (18.1)
Total	94 (100.0)

**Figure 1:**
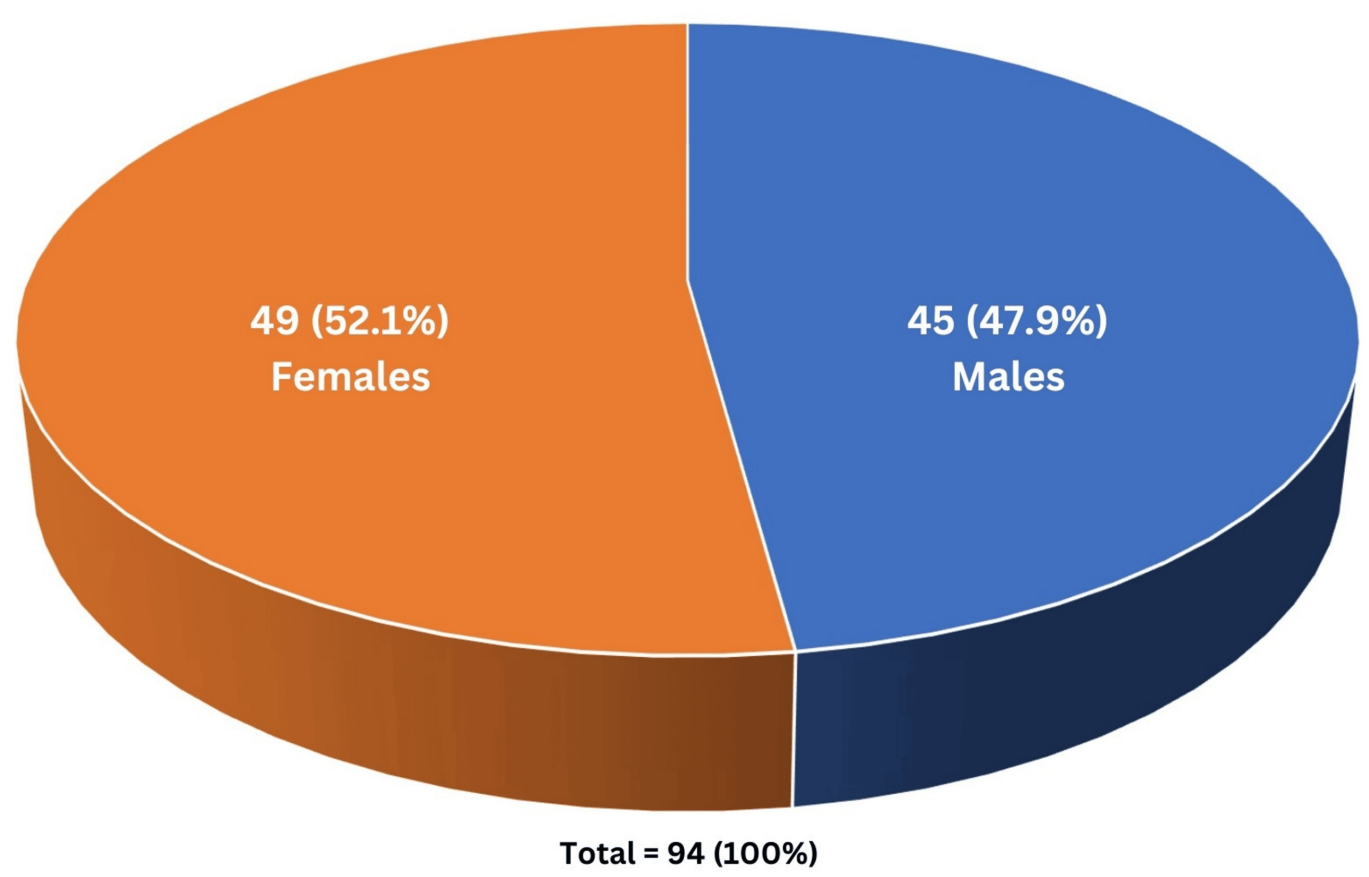
Patient distribution on the basis of gender. Of the 94 cases, 45 (47.9%) were male and 49 (52.1%) were females.

Among all the patients included in this study, 49 (52.1%) patients were females and 45 (47.9%) patients were males (Figure 1).

The mean TLC was 19.49 ± 8.12 x 10^3^/µL. The mean CRP level was 81.19 ± 56.33 mg/dL. The mean nCD64 MFI expression was 197.26 ± 79.56. As shown in Table 3, the TLC was <4.0 x 10^3^/µL in 1 (1.1%) patient, 4.0 x 10^3^/µL to 11.0 x 10^3^/µL in 15 (16%) patients, and >11.0 x 10^3^/µL in 78 (83%) patients.

**Table 3:** Patient distribution on the basis of total leukocyte count.

Total leukocyte count (x10^3^/uL)	Frequency (%)
<4.0	1 (1.0)
4.0 to 11.0	15 (16.0)
>11.0	78 (83.0)
Total	94 (100.0)

As shown in Table 4, CRP level was high in 81 (86.2%) patients and normal in 13 (13.8%) patients.

**Table 4:** Patient distribution on the basis of C-reactive protein level.

CRP level (mg/dL)	Frequency (%)
High	81 (86.2)
Normal	13 (13.8)
Total	94 (100.0)

**Figure 2:**
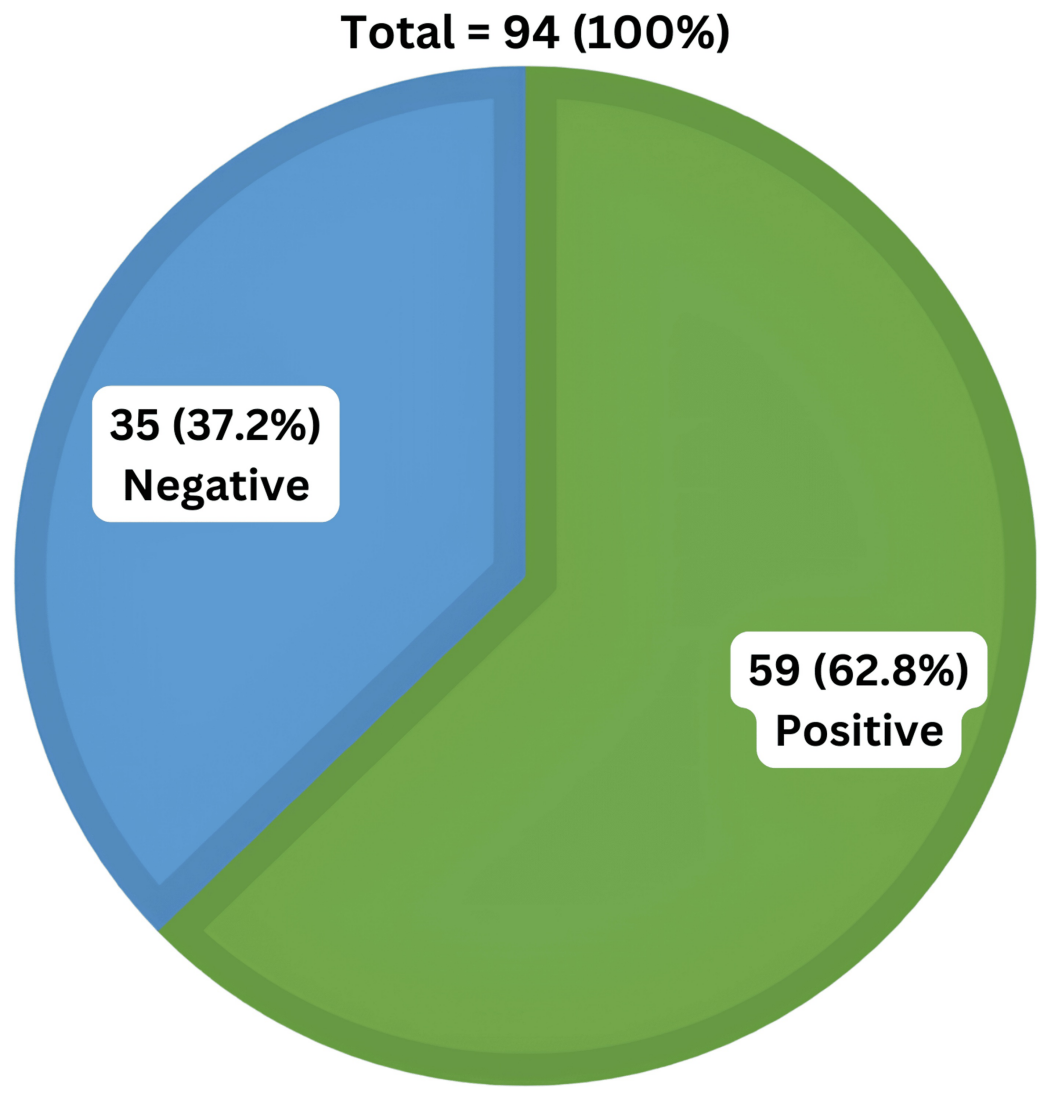
Patient distribution on the basis of blood culture reports. Blood culture test was positive in 59 (62.8%) of 94 cases and negative in 35 (37.2%).

As shown in Figure 2, nCD64 expression was significantly higher (p=0.001) in patients with positive blood cultures, i.e., 59 (62.8%) cases, supporting its role as a reliable marker for bacterial infections.

**Figure 3:**
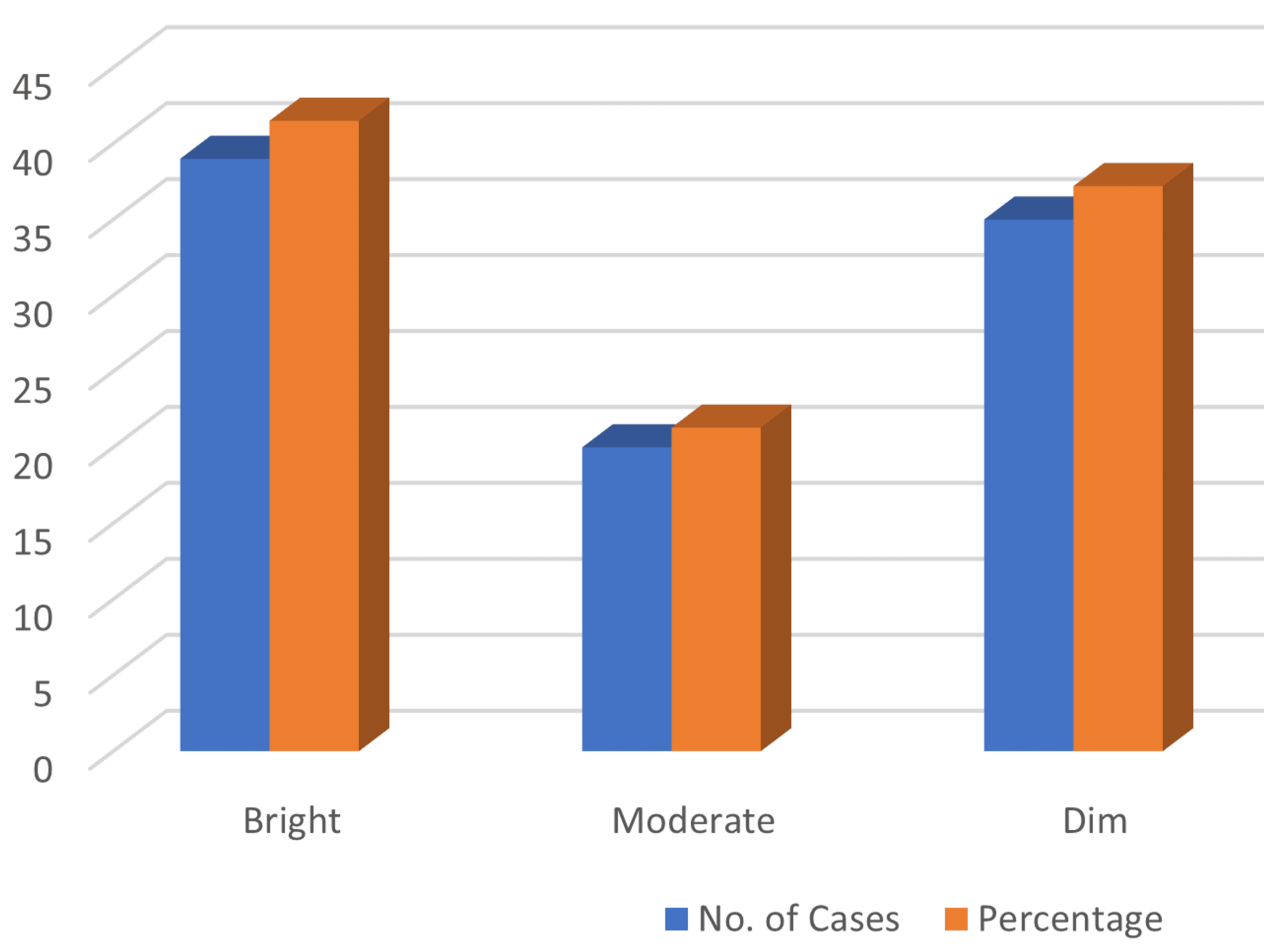
Patient distribution based on nCD64 expression. The x-axis represents nCD64 expression, and the y-axis represents the no. of cases.

Based on expression of nCD64, all 94 samples were categorized as dim, moderate, and high expression based on scattergram intensity. Based on this qualitative nCD64 expression, bright expression was seen in 39 (41.5%) patients, dim expression in 35 (37.2%) patients, and moderate expression in 20 (21.3%) patients, as represented in Figure 3.

Out of 35 patients who had negative blood culture, the nCD64 expression was moderate in 2 (10%) patients and dim in 33 (94.3%) patients. Out of 59 patients who had positive blood culture, the nCD64 expression was bright in 39 patients, medium in 18 patients, and dim in 2 (5.7%) patients. The correlation between nCD64 expression and blood culture was found to be statistically significant (p=0.001).

Of the patients, 39 had bright nCD64 expression, out of which all 39 (100%) patients had a high CRP level. Out of 20 patients who had medium nCD64 expression, all 20 (100%) patients had a high CRP level. Out of 35 patients who had dim nCD64 expression, 22 (62.9%) patients had a high CRP level and 13 (37.1%) patients had a normal CRP level. The correlation between nCD64 expression and CRP level was statistically significant (p=0.001).

In terms of TLC, out of 39 patients 1 (2.6%) patient had a TLC of 4.0 x 10^3^ to 11.0 x 10^3^/µL and 38 (97.4%) patients had a TLC of >11.0 x 10^3^/µL. Out of 20 patients who had medium nCD64 expression, 1 patient (2.9%) had TLC < 4.0 x 10^3^/µL, 12 (34.3%) patients had TLC between 4.0 x 10^3^ to 11.0 x 10^3^/µL, and 7 (62.9%) patients had TLC >11.0 x 10^3^/µL. The correlation between nCD64 expression and TLC was statistically significant. (p=0.002)

The correlation of nCD64 expression with blood culture sensitivity test, CRP level, and TLC, along with their p-values, is presented in Table 5.

**Table 5:** Patient distribution on the basis of blood culture sensitivity test, C-reactive protein, and total leukocyte count, and their correlation significance with nCD64 expression. *Refers to significant p-value (<0.05).

nCD64 expression	Blood culture sensitivity test		C-reactive protein (mg/dL)		Total leukocyte count (x10^3^/µL)		
-	Positive	Negative	High (>10.0)	Normal (1.0-10.0)	High (>11.0)	Normal (4.0-11.0)	Low (<4.0)
Bright	39 (66%)	00 (0%)	39 (48%)	00 (0%)	38 (49%)	01 (7%)	00 (0%)
Medium	18 (31%)	02 (6%)	20 (25%)	00 (0%)	22 (28%)	12 (79%)	01(100%)
Dim	02 (3%)	33 (94%)	22 (27%)	13 (100%)	18 (23%)	02 (14%)	00 (0%)
Total	59	35	81	13	78	15	01
p-Value	0.001*		0.001*		0.002*		
-	94 (100%)		94 (100%)		94 (100%)		

**Figure 4:**
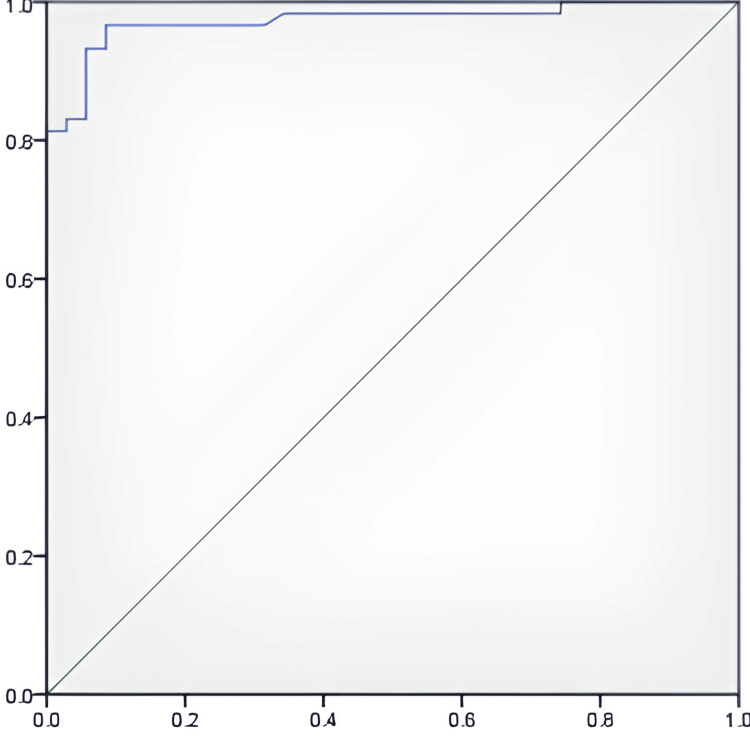
ROC curve to detect sepsis with nCD64 expression. The x-axis represents specificity, and the y-axis represents sensitivity. ROC, receiver operator characteristic

ROC analysis revealed the area under the curve (AUC) for nCD64 to be 0.973 (Figure 4). This indicates outstanding predictive accuracy and is statistically significant (p=0.001). The best cutoff value identified for predicting sepsis is 147. At this threshold, nCD64 achieves a sensitivity of 93.2%, meaning it correctly identifies 93.2% of true sepsis cases. Additionally, it has a specificity of 91.4%, indicating that it accurately recognizes 91.4% of cases that are not sepsis.

Therefore, the study found that nCD64 had higher sensitivity and specificity compared to traditional sepsis markers such as CRP and TLC, potentially offering a faster and more accurate diagnostic tool for clinicians.

## Discussion

Our findings are consistent with previous studies that suggest nCD64 is a more reliable marker for sepsis diagnosis. Unlike CRP, which can be elevated in non-infectious inflammatory conditions, nCD64 expression is more specific to bacterial infections.

Bacterial infections are becoming an increasingly serious issue that cannot be disregarded due to the rise in antibiotic resistance. Bacterial culture takes a long time and yields results with low sensitivity; thus, there is a great demand for quick indicators that could help identify bacterial infections and guide the administration of antibiotic medication. Therefore, the novel indicator nCD64 index was the focus of this investigation in the diagnosis of adult sepsis.

A study by Hu et al. observed that the patients had a mean age of 63.59 ± 13.82 years with the majority of male patients, i.e., 52.94% [9]. In our present research, the mean age of the patients was 47.82 ± 17.99 years. Most (30.9%) patients belonged to the age group of 19 to 35 years in our study. Another study by Farias et al. included 93 patients between 15 and 95 years of age, with a higher male preponderance (52.7%) [16]. Conversely, the present study showed a higher female preponderance (52.1%).

In the present study, the mean TLC was 19.49 ± 8.12 x 10^3^/µL, mean CRP level was 81.19 ± 56.33 mg/dL, and the mean nCD64 MFI expression was 197.26 ± 79.56. These values were in line with the findings of Farias et al., who observed a CRP level of 126.5 mg/dL (15.8-346) and TLC of 15.16 (9.84-21.59) x 10^3^/µL [16]. Recognizing sepsis is critical. nCD64 has been shown in numerous studies to have good sensitivity and specificity for detecting sepsis when compared to other global indicators of inflammation as PCT and CRP [17,18].

Patients with suspected sepsis due to either a bacteremia or fungemia can benefit from blood cultures to help with their diagnosis. Blood cultures should ideally be obtained before starting the proper anti-bacterial or anti-fungal treatment. nCD64 expression on neutrophils was bright in 39 (41.5%) patients, dim in 35 (37.2%) patients, and moderate in 20 (21.3%) patients in our study. The possibility that blood culture will be positive rises in patients who exhibit clinical signs of illness.

Ghosh et al. in their study reported the mean TLC as 17 (13‑21) x 10^3^/µL and mean nCD64 MFI as 163 (143‑183). In the present study, 83% patients had TLC > 11.0 x 10^3^/µL, and among these patients, 49% had bright nCD64 levels and 28% had moderate nCD64 levels [19].

The rate of false-positive blood culture increases in a patient with very low likelihood of bacteremia. Chang et al. found that in emergency departments with high volume of patients, those with critical illness, those with end-stage renal disease, and the elderly were more likely to have blood culture contamination [20]. The correlation between nCD64 expression and blood culture was statistically significant (p=0.001).

The correlation between nCD64 expression with CRP level and TLC was statistically significant in our study. This might be explained by the fact that only patients with microbiologically verified infections were included in the infected group, which resulted in a higher cutoff, and by the fact that individuals with fungal or viral infections are anticipated to express nCD64 more than usual. A significant correlation of nCD64 expression with CRP level and TLC was also found in studies by Matsui et al. and Jalava-Karvinen et al. [21,22].

In the present study, the AUC for nCD64 was 0.973, which indicated outstanding predictive accuracy and was statistically significant (p=0.001). Our findings indicate that nCD64 expression is a valuable diagnostic marker for sepsis diagnosis upon admission, with a sensitivity of 93.2% and specificity of 91.4%. These results are in line with previous studies by Cardelli et al., Chang et al., and Gibot et al. in critically ill adult patients [17,18,23].

In a study by Cardelli et al., nCD64 expression had 96% sensitivity and 95% specificity (AUC = 0.97) for detecting bacteremia among 112 patients selected using systemic inflammatory response syndrome (SIRS) criteria [17]. Chang et al. reported that nCD64 expression discriminated severe sepsis or septic shock from SIRS with 89% sensitivity and 96% specificity (AUC = 0.93) in 66 patients with respiratory failure [18]. In a more recent study by Gibot et al., nCD64 expression had 84% sensitivity and 95% specificity (AUC = 0.97) in identifying sepsis among 300 critically ill patients [23]. These results, and ours, contrast with the lower AUC (0.80) and sensitivity of nCD64 expression (63%) reported by Gros et al., although the reported specificity was high (89%) [24]. The inclusion of patients with viral or fungal infections and those with microbiologically confirmed infection could result in a higher cutoff value [13].

Previous studies by Cid et al. and Groselj-Grenc et al. have indicated that nCD64 expression is highly correlated to the presence of infection or inflammatory process [13,25]. Patients with bacterial infections usually have a large increase in nCD64 expression. The source of this notable rise, however, is still up for debate. Some argue that it may be attributed to either an increase in nCD64 expression or an increase in molecule surface density.

Limitations of this study

The present study is a single centre study, which could have limited the applicability of our findings to other environments. Secondly, sepsis is a condition with a significant degree of heterogeneity rather than being just a singular disease entity. Misclassifications might have happened even when we used post hoc diagnosis to reduce the likelihood of them.

## Conclusions

nCD64 has proven to be a highly sensitive and specific biomarker, outperforming TLC, CRP levels, and blood culture sensitivity tests in diagnosing sepsis. It offers a quick and efficient method that can complement other biomarkers to improve diagnostic accuracy. The use of nCD64 in clinical practice holds great potential in streamlining the sepsis diagnostic process. Despite these promising results, further multicentric studies are necessary to validate these findings. Incorporating nCD64 testing could play a crucial role in enhancing diagnostic precision in sepsis cases.
